# Photo-Electrochemical Sensing of Dopamine by a Novel Porous TiO_2_ Array-Modified Screen-Printed Ti Electrode

**DOI:** 10.3390/s18103566

**Published:** 2018-10-21

**Authors:** Francesco Tavella, Claudio Ampelli, Salvatore Gianluca Leonardi, Giovanni Neri

**Affiliations:** 1 Department of Chemical, Biological, Pharmaceutical and Environmental Sciences, University of Messina, I-98166 Messina, Italy; ftavella@unime.it (F.T.); ampellic@unime.it (C.A.); 2 Department of Engineering, University of Messina, I-98166 Messina, Italy; leonardis@unime.it

**Keywords:** TiO_2_, dopamine, photo-electrochemical sensors

## Abstract

In this paper, the development of a nanoporous TiO_2_ array-modified Ti electrode for photo-electrochemical (PEC) sensing of dopamine (DA) is reported. A porous TiO_2_ array-modified electrode was fabricated from the controlled anodic oxidation of a Ti working electrode of commercial screen-printed electrodes (SPE). The anodization process and the related morphological and microstructural transformation of the bare Ti electrode into a TiO_2_/Ti electrode was followed by scanning electron microscopy (SEM) and UV-visible reflectance spectroscopy (DR-UV-Vis). The modified electrode was irradiated with a low-power (120 mW) UV-Vis LED lamp (λ = 400 nm) and showed good performance for the detection of DA with a large linear response range, a sensitivity of 462 nA mM^−1^ cm^−2^, and a limit of detection of 20 µM. Moreover, it showed higher photocurrents in the presence of DA in comparison to some foreign species such as ascorbic acid, uric acid, glucose, K^+^, Na^+^, and Cl^−^. Thus, this proposed low-cost photo-electrochemical sensor, with the advantage of very simple fabrication, demonstrates potential applications for the determination of dopamine in real samples.

## 1. Introduction

The sensitive determination of biomolecules using electrochemical sensors based on nanostructured sensing materials has acquired high importance for disease diagnoses and drug screening [1,2]. In this respect, one widely investigated subject in the field of electroanalysis is the determination of neurotransmitters [3,4]. Among these substances, dopamine (DA) assumes a key role in ensuring inter-neuronal communication in the human central nervous system [5]. It is vital to many neuronal functions like memory, learning, cognition, behaviour, attention, emotion, and movement. Abnormal responses of dopamine may cause several diseases like epilepsy, schizophrenia, and Parkinson’s disease [5]. This explains the huge investigative efforts deployed during the last three decades for the determination of dopamine.

Determination of dopamine by electrochemical sensors has largely been pursued in recent years [6,7,8]. However, dopamine and its oxidation products are very reactive and result in the formation of polydopamine, which has a strong adhesion capability to electrode surfaces [9]. To limit electrochemical fouling by the above-mentioned reactive species, electrochemical sensors with a photoactive sensing layer have been proposed [10,11,12]. These sensors also show higher sensitivity, as well as the ability to operate with lower background signals and noise with respect to conventional sensors. For an effective photoactive material in the fabrication of electrodes for photo-electrochemical determination of dopamine, those based on titania (TiO_2_) nanostructures appear to be the most suitable [10,11,13,14,15,16,17].

To fabricate simple photo-electrochemical sensors, the working electrode of screen-printed electrodes (SPE) can be modified by depositing a TiO_2_-sensitive layer over its surface. Previously, various deposition methods have been proposed for the fabrication of photo-electrochemical TiO_2_ electrodes using different electrode materials such as carbon, gold, copper, ITO, and so on [18,19]. However, the many concerns arising from the scarce reproducibility of the deposition of the sensing layer or its adhesion to the substrate, etc., can limit their practical use.

The anodic oxidation of Ti foils in F^−^-containing electrolytes to synthesize size-controlled TiO_2_ nano-dimensional arrays (such as TiO_2_ nanopores or nanotubes) is an attractive method for its potential to up-scale and its high degree of control over size and morphology [20,21,22]. These TiO_2_/Ti layers have been used as electrochemical sensors [23] and in photo-activated gas sensor applications because of the advantages of lower cost, higher surface area ratio, stability, and environmental friendliness [24]. Photo-electrochemical (PEC) systems based on modified TiO_2_ electrodes have also been developed. Ojani et al. have fabricated a Ti/TiO_2_ electrode; in this case, a titanium foil was employed as the substrate and subjected to an anodization process [25].

However, to the best of our knowledge, no study has reported modification of Ti working electrodes of commercial screen-printed electrodes. For this purpose, we exploited the advantageous photo-electrochemical features of nanostructured porous TiO_2_ to be used in the electrocatalytic detection of dopamine. For the fabrication of this novel modified electrode, nanopore arrays of TiO_2_ were grown in situ on Ti working electrodes by an anodization process. Finally, the efficiency of this electrode in the determination of low concentrations of dopamine was investigated using a low-power Light-Emitting Diode (LED). The combination of cost-effective and low power operation of the device response means that this device could be applicable for practical use in the biomedical field.

## 2. Materials and Methods

### 2.1. TiO_2_ Synthesis

To fabricate nanoporous TiO_2_-modified electrochemical sensors, commercial Ti screen-printed electrodes (Dropsens DRP-TI10, supplied by Metrohm, Asturias, Spain) consisting of a titanium working electrode (Ti WE, 4 mm in diameter), a carbon counter electrode, and a silver pseudo-reference electrode were used. Nano-ordered pores of titania were generated over Ti WE by controlled anodic oxidation using an ethylene glycol (EG) solution (containing 0.3 wt.% NH_4_F and 2 wt.% water) as the electrolyte. The anodic oxidation was carried out in a stirred electrochemical cell operating at room temperature. A DC power supply (Agilent E3612A, supplied by RS Components S.p.A., Milan, Italy) was used to drive the anodization, and a multimeter (Keithley 2000/E, supplied by Tektronix Srl, Padua, Italy) was employed to record the resulting current. The applied potential (50 V) was maintained at a constant for 30 min during the anodization. Further details on the equipment and procedure have been reported elsewhere [26]. However, we started from a pure Ti foil (3.5 cm diameter, 0.025 mm thickness), which was oxidized in a fluoride-based electrolyte. The novelty of the present work, instead, is in the modification by anodic oxidation of a commercial screen-printed sensor containing a small plate of Ti (4 mm diameter) acting as the working electrode. The different purities and sizes of the Ti substrates influenced the whole anodization process, and many efforts were made to adapt the procedure to this small Ti electrode.

### 2.2. Characterization

The morphology and microstructure of bare Ti and modified TiO_2_/Ti electrodes were observed using a ZEISS 1540XB FE SEM (Carl Zeiss AG, Oberkochen, Germany) instrument operating at 5 kV. The chemical composition of the oxide layers was determined by energy dispersive X-ray spectroscopy (EDX).

An UV/Vis spectrometer (Jasco V-570 PerkinElmer Lambda 1050, Easton, MD, USA) equipped with an integrating sphere for solid samples was employed for diffuse reflectance measurements in air using BaSO_4_ as the reference.

### 2.3. Photoelectrochemical Tests

The modified screen-printed electrodes were tested in a homemade apparatus designed for photo-electrochemical studies under both dark and UV-Vis light (400 nm) provided by cost-effective and low powered commercial LEDs (120 mW). All the electrochemical experiments were performed inside a 5 mL cell at room temperature and in the presence of air above the analyte solution. Potential and current were recorded by means of a Dropsens µStat 400 potentiostat using the same screen-printed counter and reference electrodes of the commercial device. Cyclic voltammetries (CV) were performed at a scan rate of 100 mV s^−1^ in the potential range between 0 and 1 V.

## 3. Results and Discussion

### 3.1. Synthesis and Characterisation

**Figure 1:**
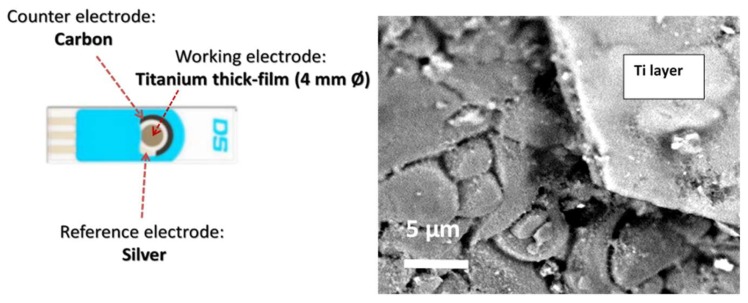
Diagram of the electrochemical sensing platform used (**left**). SEM image showing a cross section of the thick titanium layer (about 1.5 µm) on the alumina substrate (**right**).

Figure 1 shows a diagram of the electrochemical sensing platform used. In order to fabricate the modified electrode, the Ti working electrode of the screen-printed electrode was subjected to an anodisation process under the conditions reported in Section 2 (50 V, 30 min).

**Figure 2:**
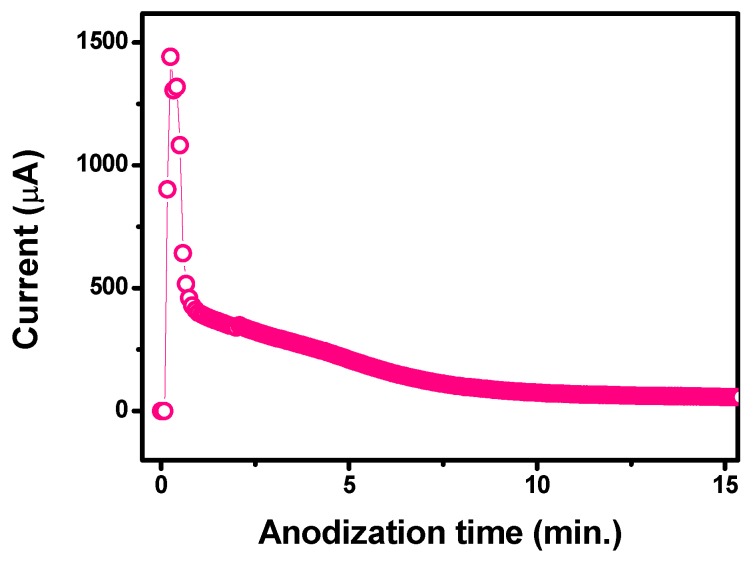
Current–time curve obtained during anodic oxidation of the Ti working electrode.

The anodization process was followed in real time and the current across the working electrode was acquired. The current–time curve obtained during the entire anodising process is shown in Figure 2. Three steps can be clearly identified. Initially, the current was very low, indicating the presence of an insulating TiO_2_ layer on the surface of Ti WE; then, the current rapidly increased to some extent because of the etching of the thin TiO_2_ layer due to the presence of F-based ions in solution, and thus exposing the Ti metal. After this initial process, by increasing the anodising time, the Ti-exposed surface was readily converted into a thin TiO_2_ layer, leading consequently to a fast current decrease. As the anodization proceeded into bulk, i.e., as the TiO_2_ array nanostructure size extended in the axial direction, the current decreased again, but at a lower rate. Beyond this point, the current was almost steady, which indicated the almost complete growth of the nanopore arrays as a result of the equilibrium between the electrochemical formation of TiO_2_ at the pore bottom and the chemical dissolution of this TiO_2_ in the F^−^ ion solution.

From a practical point of view, this approach is much simpler and more efficient than other post-processing procedures which attempt to attach the photo-electrochemical active TiO_2_ nanostructures onto the bare electrodes. As in previous reports described in Section 1, the photo-electrochemical active TiO_2_ nanostructures are attached onto the bare electrodes by post-processing procedures, i.e., the TiO_2_ nanostructures are formed in a separate anodization step of Ti foils, and then they are successively transferred onto the bare electrodes. This procedure is, of course, complicated and leads to only a physical attachment of the photo-electrochemical active TiO_2_ nanostructures to the bare electrodes with an evident limitation of the electron transfer across the interface layer.

**Figure 3:**
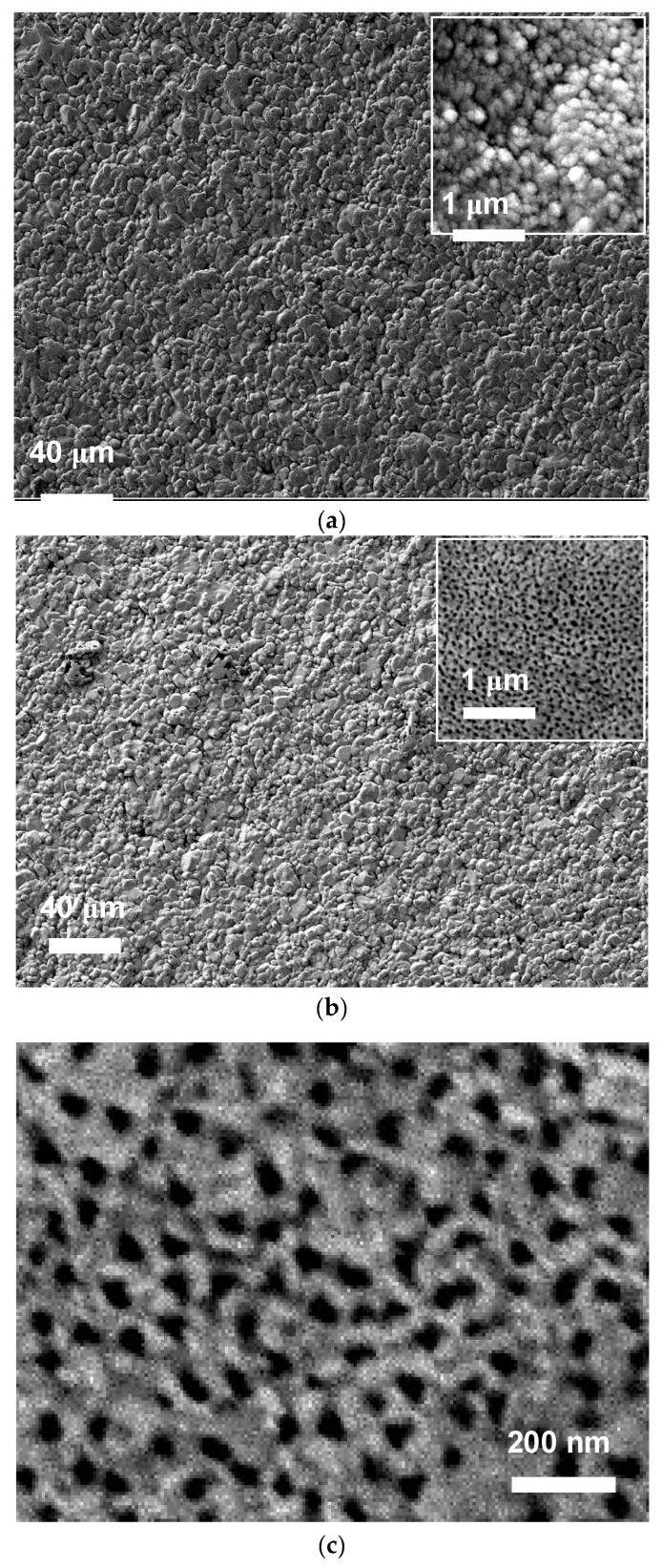
Morphology of Ti screen-printed electrodes (**a**) before and (**b**) after the anodic oxidation treatment. (**c**) High magnification image of TiO_2_ grown on the Ti electrode showing the highly porous array structure.

To obtain information on the morphological modifications that occurred after the anodization process, the surfaces of both the bare and anodised Ti electrodes were observed by SEM (Figure 3). It is well known that the characteristics of the obtained TiO_2_ nanostructures on Ti foils, such as size, shape, packing density, and length, depend on several synthesis parameters: electrolyte type, applied voltage, pH, and anodization time, etc. [26,27]. However, so far no study has been undertaken to investigate the modifications that occur by using the thin Ti film of commercial electrodes as a substrate. Figure 3a,b shows the morphology of the Ti screen-printed electrode before and after treatments carried out at high voltage (50 V) in the presence of fluoride anions and ethylene glycol. A high magnification image of the nanoporous TiO_2_ arrays on the Ti electrode is presented in Figure 3c, showing the highly porous array structure. These nanoporous TiO_2_ arrays were characterised by the presence of pores of irregular shape that were approximately 50 nm in diameter.

EDX analysis (not shown) of the bare electrode indicated the presence of Ti and O, the latter coming from the thin amorphous TiO_2_ top layer already present on the Ti electrode surface. After anodization of the bare Ti electrode, O peaks increased in intensity and the Ti/O atomic ratio of the modified working electrode approximately equalled the TiO_2_ stoichiometric ratio.

The treatment clearly modified the working electrode surface and formed well-developed TiO_2_ nanoporous arrays on the entire electrode surface area. The resulting nanoporous array structure appeared to be well-adhered to the underlying Ti substrate. This is a clear advantage derived from the electrochemical cycling, which guarantees mechanical stability to this sensor. Moreover, this significant improvement of adhesion between the TiO_2_ nanoporous arrays and the underlying Ti metal layer can also be beneficial in increasing the photo-conversion efficiency, which greatly depends on the geometric roughness factor of the photocatalytic surface layer and its adhesion to the underlying metal layer.

The resulting extended nanoporous array oxide layers will not only contribute to greatly enhancing the sensitivity due to their higher surface area, but also provide a wide linear range for the electrochemical determination of analytes in comparison to bare electrodes with lower surface area. Lastly, in regards to their use as photo-electrochemical sensors, it can be argued that in situ growth of the photo-electrochemical active TiO_2_ nanostructures on the bare Ti electrode via the adopted anodising procedure could provide the formation of Schottky-type contacts between TiO_2_ arrays and Ti substrate, thus resulting in a structure facilitating the transport of photo-generated electrons.

**Figure 4:**
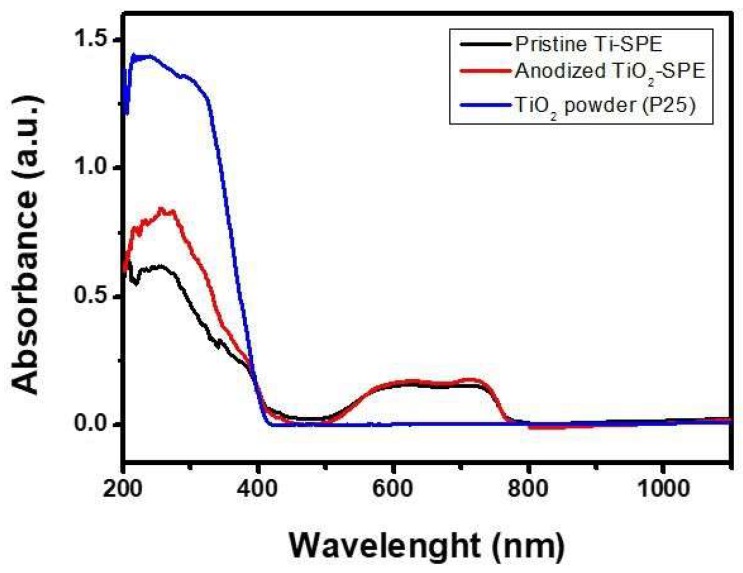
UV-Vis diffuse reflectance spectra of bare Ti-SPE and modified TiO_2_-nanoporous SPE. The spectrum of reference, TiO_2_ Degussa P25, is also shown.

Figure 4 reports the absorbance curves in the UV-vis region spectra of bare Ti-SPE and modified TiO_2_-nanoporous SPE. The spectrum of the commercial TiO_2_ P25 Degussa has also been reported for reference. The spectra are characterized by a strong absorption below 400 nm related to the typical band gap of TiO_2_. All spectra exhibited a strong absorption band centred at about 250–350 nm associated with the TiO_2_ band gap.

The spectra also evidenced a broad absorption peak in the visible region, extending from around 500 to 780 nm, except in the case of commercial TiO_2_ Degussa P25 nanoparticles. This visible light absorption could be due to a structural resonance effect typical of 2D-type photonic materials [28]. This effect can be seen with titanium oxide, especially for nanoporous structures, due to the positive interaction between the wavelength of irradiated light and the periodicity of the crystal structure, thus providing a wide absorption in the 500–780 nm range, as previously observed [29]. However, the modified TiO_2_-nanoporous SPE did not take advantage of this light absorption property as it generated a photocurrent when irradiated by a LED at 400 nm, as will be shown in Section 3.2.

### 3.2. Electrochemical Behaviour

**Figure 5:**
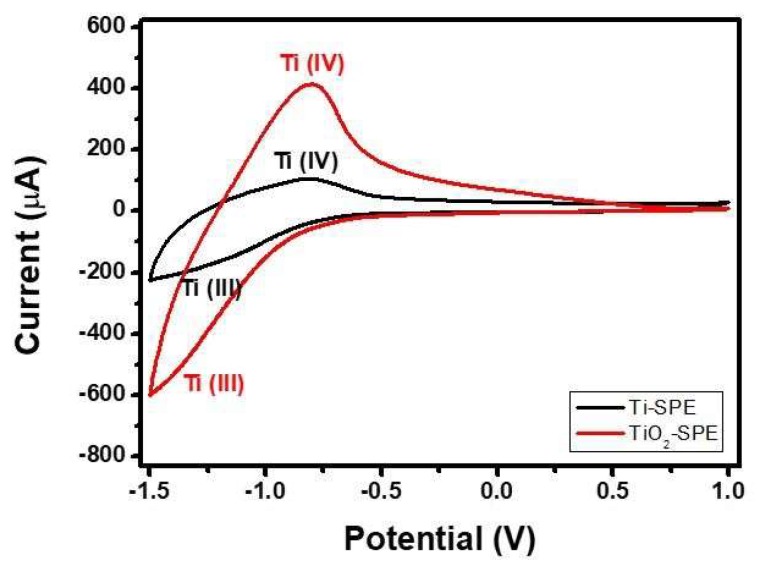
CV of bare Ti-SPE and TiO_2_-SPE electrodes in PBS (pH = 7) at 50 mV s^−1^.

To evaluate the electrochemical behaviour of the bare Ti-SPE and modified TiO_2_-nanoporous SPE, cyclic voltammetry (CV) experiments were carried out. For this purpose, the CV experiments were performed in dark in Phosphate-Buffered Saline (PBS) solution (pH 7.0). Results obtained are summarized in Figure 5. Through the analysis of the cyclic voltammograms of both electrodes at negative potentials, it is possible to distinguish a pair of peaks characteristic of the Ti(III)/Ti(IV) transformation of titanium electrodes [30].

The reversible redox reaction involved can be described by Equation (1) [31]:

TiOOH ↔ TiO_2_ + H^+^ + e^−^ (1)

Furthermore, the large area of the cycle for modified TiO_2_-nanoporous SPE, which results from a large charge density, suggests an increase of the specific surface area of the electrode as an effect of the high porous structure after the anodization process of the Ti electrode [32].

**Figure 6:**
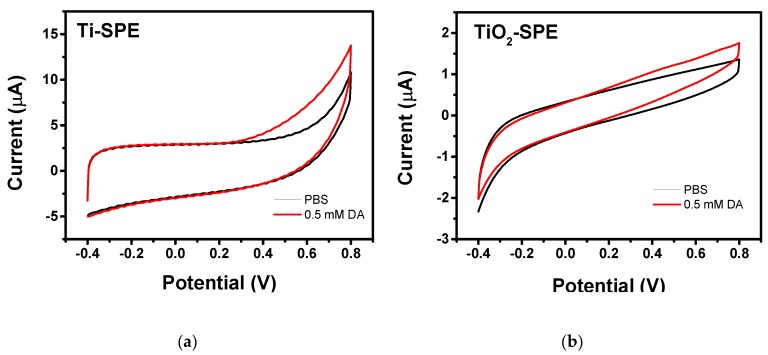
CVs of (**a**) bare Ti-SPE and (**b**) TiO_2_-nanoporous SPE in PBS (pH = 7.0) in the absence and presence of 0.5 mM DA. Scan rate is 50 mV s^−1^.

The electrocatalytic activity towards dopamine oxidation has also been investigate for both the electrodes. Figure 6a,b shows the cyclic voltammograms of bare Ti-SPE and modified TiO_2_-nanoporous SPE recorded in dark and in the absence and presence of 0.5 mM DA in pH 7 phosphate buffer solution, respectively. No evident anodic/cathodic peaks were observed on the CV curves registered with both bare Ti-SPE and modified TiO_2_-nanoporous SPE in neither the absence nor the presence of DA. Only a slight increase of the anodic current could be observed, with an onset potential of 0.2 V, when dopamine was present in the solution. These results can be interpreted with the assumption that both the electrodes show a poor electrocatalytic activity towards DA oxidation in dark conditions.

Both bare Ti-SPE and modified TiO_2_-nanoporous SPE did not give appreciable photocurrents under UV irradiation on CVs. However, the same behaviour was also noted in the presence of DA. This finding is likely due to the very low power of the LED used, which are not able to generate large currents in comparison to the capacitive currents typical of CV analyses.

### 3.3. Photo-Electrochemical Tests

**Figure 7:**
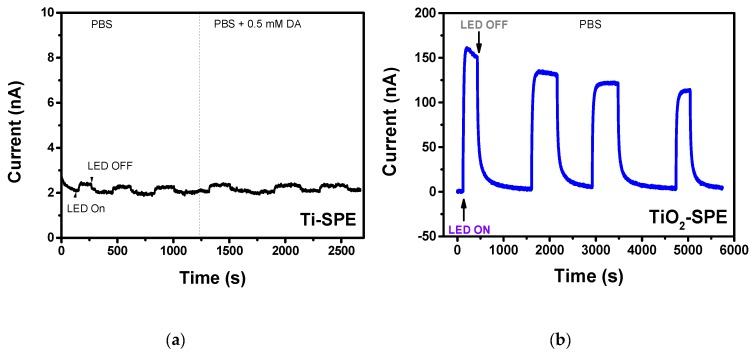
(**a**) Photocurrent signal of the bare Ti-SPE registered at V Ag/AgCl = 0 V under pulses of UV LED light (400 nm) in the absence of and in the presence of different concentrations of dopamine. (**b**) Photocurrent signal of the modified TiO_2_ electrode in the same conditions in the absence of dopamine.

Photo-electrochemical experiments were performed by chronoamperometric methods at an applied potential of 0 V where, according to CV analysis, any oxidation process was observed. Figure 7a shows the amperometric response of bare Ti-SPE when exposed to pulses of UV LED light (400 nm) in the absence and presence of 0.5 mM DA. As expected, only a very small photocurrent generated by the formation of the electron-hole was observed when LED light was switched on. This effect could be due to the formation of a thin layer of TiO_2_ on the surface of the Ti electrode. A similar increase of the photocurrent was observed in the presence of DA, with an intensity close to that recorded in the presence of only PBS. This finding indicates definitively that the bare Ti-SPE electrode is unable to be used for the application considered here, nor is it able to be used under UV LED irradiation.

On the contrary, the modified TiO_2_-nanoporous SPE showed a large photocurrent when exposed to pulses of LED light (Figure 7b). The observed behaviour suggests that the TiO_2_-nanopore array structure acts as a photo-electrochemical active layer, facilitating the spatial separation of the photo-generated charge carriers, and therefore resulting in an enhanced photocurrent response. Although a decrease of the induced photocurrent occurred during the first pulses, this transitory effect disappeared with the successive pulses.

It is well known that DA molecules have a large affinity for low-coordinated Ti atoms at the surface of TiO_2_ [33]. Under illumination, DA/TiO_2_ charge transfer complexes can be formed and the excited electrons from DA molecules transfer directly to the conduction band of TiO_2_ and generating holes localized on the DA molecules, which further promotes spatial separation of the photo-generated charges, thus significantly enhancing the PEC performance [33]. This behavior has been exploited for developing a PEC sensor for this analyte, which is of considerable interest in the biomedical field.

**Figure 8:**
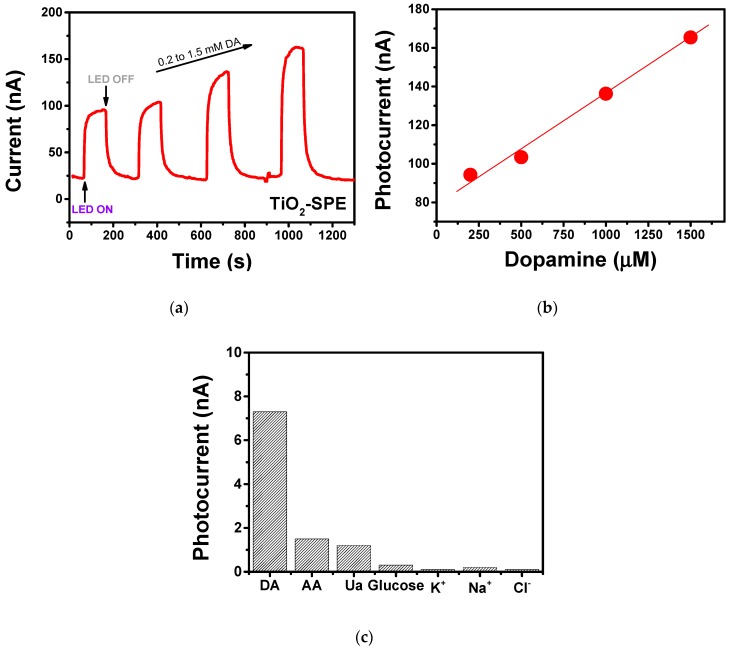
(**a**) Photocurrent signal of the modified photo-electrochemical sensor registered at V Ag/AgCl = 0 under pulses of UV LED light (400 nm) in the presence of different concentrations of dopamine. (**b**) Calibration curve. (**c**) Photocurrent variation registered in the presence of 0.5 mM of DA and some foreign species.

The electroanalytical properties of the modified TiO_2_-nanoporous SPE in the determination of dopamine in PBS are shown in Figure 8. A clear enhancement of the response to an increase of dopamine concentration was observed (Figure 8a). The corresponding calibration curve is reported in Figure 8b, showing the wide linear range of the sensor extending up to 1500 µM. The sensitivity of the modified electrode for dopamine was calculated to be around 462 nA mM^−1^ cm^−2^, and the limit of detection (LOD), at a signal-to-noise ratio of 3, has been estimated as 20 µM. Tests also showed that the presence of some foreign species such as ascorbic acid, uric acid, glucose, K^+^, Na^+^, and Cl^−^, had little or no effect on the photocurrent (Figure 8c), indicating good selectivity towards the target analyte.

Given that self-organized and well-aligned TiO_2_ nanostructures have been reported to exhibit highly efficient PEC responses compared to nanoporous arrays due to more direct path for charge carriers transport and large surface areas for light harvesting [34], we have planned further studies in order to optimize the experimental conditions for the formation of aligned TiO_2_ nanotubes on the Ti-SPE platform.

Table 1 compares the performances of photo-electrochemical sensors based on TiO_2_. Although our sensor has lower performance, it must be considered that in this case a 120 mW LED was used which has very low power in comparison to other cases. In addition, it is worth pointing out that the optimization of the optical power radiated on the electrode surface by means of a focusing system leaves ample room for improvement.

However, further tests for long-term stability and reproducibility are needed before the implementation of the modified TiO_2_-nanoporous SPE.

## 4. Conclusions

A new and simple method to fab ricate photo-electrochemical sensors has been developed based on the modification of commercial screen-printed Ti electrodes by controlled anodic oxidation with in situ growth of titania nanopore arrays. The modified TiO_2_ photoelectrode was prepared by anodising the Ti electrode layer in HF aqueous solution. Characterisation results showed that the TiO_2_-array layer formed on the Ti electrode was characterised by the presence of nanopores, which greatly increased the surface area of the bare electrode. The modified TiO_2_ photoelectrode was tested for the determination of dopamine assisted by low-power UV-Vis LED at 0 V vs. Ag/AgCl, and the results showed good sensitivity, a low limit of detection, and a wide linear range. Additional advantages, such as the simplicity, speed of fabrication, low cost, and, most importantly, the employment of a small-sized and low-consumption UV-Vis LEDs as the illumination lamp, promise that the sensor developed could be suitable for practical use.

## Figures and Tables

**Table 1:** Comparison of TiO_2_-based photo=electrochemical dopamine sensors.

Electrode	Range (μM)	Light Source	Sensitivity (nA μM^−1^ cm^−2^)	Electrode Area (cm^2^)	Detection Limit (μM)	Ref.
Graphene–TiO_2_	0.02–105	250 W Xe lamp	2140	0.07	0.006	[13]
TiO_2_ NTs	0.001–25	300 W Xe lamp	1340	--	0.00015	[15]
TiO_2_ NPs	5–200	30 W LED	0.013	2.4	2	[16]
CuTsPc/TiO_2_	4–810	20 W LED	3.7	0.8	0.5	[17]
Graphene-C_3_N_4_/TiO_2_	0.1–50	150 W Xe lamp	210 (nA/μM)	--	0.02	[35]
TiO_2_ nanopore array	200–1500	120 mW LED	0.462	0.125	20	This work
